# Comparison Between Total Hip and Knee Arthroplasty on Short-Term Performance-Based Outcomes and Factors Associated with the Improvement of Gait Function During Post-Acute Inpatient Rehabilitation

**DOI:** 10.3390/jcm13216381

**Published:** 2024-10-24

**Authors:** Kenichi Kawaguchi, So Kuwakado, Hiroshi Otsuka, Akemi Sakugawa, Masanori Takahashi, Taiji Oda, Goro Motomura, Satoshi Hamai, Yasuharu Nakashima

**Affiliations:** 1 Department of Rehabilitation Medicine, Kyushu University Hospital, Fukuoka 812-8582, Japan; kuwakado.so.335@s.kyushu-u.ac.jp; 2 Department of Rehabilitation Medicine, Fukuoka Mirai Hospital, Fukuoka 813-0017, Japan; hiroshi-otsuka@lta-med.or.jp (H.O.);; 3 Department of Orthopaedic Surgery, Graduate School of Medical Sciences, Kyushu University, Fukuoka 812-8582, Japan

**Keywords:** total hip arthroplasty, total knee arthroplasty, rehabilitation, gait function, knee extensor strength, pain intensity

## Abstract

**Background**: Data on the differences in functional recovery between inpatient rehabilitation for total hip arthroplasty (THA) and total knee arthroplasty (TKA) are lacking, and the factors influencing the improvement of short-term functional mobility remain unknown. In this study, we compared the short-term functional outcomes of both procedures and identified early postoperative predictors of physical function gain during post-acute rehabilitation. **Methods**: A total of 435 patients who underwent THA and TKA were included. The main outcomes were knee extension strength, the motor component of the Functional Independence Measure, Numerical Rating Scale, 10 Meter Walk Test, Timed Up and Go (TUG) test, and the Berg Balance Scale. The recovery process and rehabilitation outcomes were compared between patients with THA and TKA. Additionally, predictors related to physical performance improvement were examined for each procedure. **Results**: Patients with THA and TKA achieved significant short-term functional recovery after multidisciplinary rehabilitation (time; *p* < 0.001). However, the pain score was higher at discharge in patients with TKA (*p* < 0.001). Age (β: −0.264, *p* = 0.009) and TUG test (β: −0.884, *p* < 0.001) in THA, and non-operated knee extension strength (β: 0.234, *p* = 0.016) and TUG test (β: −0.783, *p* < 0.001) in TKA were significant early postoperative predictors of functional mobility. **Conclusions**: Multidisciplinary rehabilitation was beneficial for functional improvement in patients with THA and TKA despite persistent pain at discharge after TKA. Baseline functional levels in both groups and non-operated knee extension strength in TKA can be useful performance-based predictors of short-term gait function improvement.

## 1. Introduction

Total hip arthroplasty (THA) and total knee arthroplasty (TKA) are highly successful and cost-effective procedures for relieving chronic joint pain and improving function in patients with end-stage osteoarthritis [1,2,3,4,5,6,7]. Despite these effects, only a few researchers have compared the physical outcomes of patients who undergo THA and TKA. Previous studies have suggested that primary THA offers superior short-term outcomes for 6 months during the postoperative period compared to primary TKA [1,2,3]. This indicates that patients who underwent TKA have a slower rate of recovery during the early postoperative phase. Other authors have reported similar outcomes between patients who underwent THA and those who underwent TKA at 3–6 months postoperatively [4,5,6]. The differences in recovery regarding physical function [4,5,6], pain [6], and health-related quality of life [4] in the short term remain a topic of debate. However, the greatest improvement in motor function for patients after THA and TKA is typically observed in the first few months postoperatively [8]. Therefore, understanding the recovery process of each procedure is essential for effective post-acute rehabilitation interventions.

Intensive postoperative rehabilitation is important for improving physical function in the early postoperative period. A previous study indicated that multidisciplinary rehabilitation after total joint arthroplasty is associated with improved activity and participation outcomes [9]. Thus, it is important to accurately assess the effectiveness of rehabilitation using the appropriate evaluation tools to determine whether independent activities of daily living (ADL) can be sufficiently achieved during the post-acute period. Rehabilitation outcomes can be measured using physical performance tests, such as the Timed Up and Go (TUG) test, to assess the gain of function in the short and middle term following THA and TKA [10,11,12]. In the several months after surgery, performance-based assessments can capture the actual change in functional performance, whereas assessments using patient-reported outcome measures tend to overestimate outcomes because of the beneficial impact of arthroplasty and significant pain relief following surgery [11,13,14]. Previous studies have suggested that preoperative and early postoperative physical function can be the most useful predictors of mobility during the first 6 months following THA and TKA [5,15]. A detailed assessment of the effect of multidisciplinary rehabilitation on the outcomes of THA and TKA in the short-term postoperative recovery process has been minimally discussed in the literature. The identification of patient characteristics and clinical predictors associated with functional mobility improvement in patients with THA and TKA is important for facilitating appropriate care and management during inpatient rehabilitation.

This study aims to analyze improvements in physical function in patients who underwent THA and TKA and to determine the factors that influence short-term functional mobility after each procedure in post-acute rehabilitation settings. We hypothesize that the recovery of functional performance will be similar between the two groups during inpatient rehabilitation, except for pain intensity, and that early postoperative mobility will be associated with gait function at discharge.

## 2. Methods and Methods

### 2.1. Patients

This retrospective cohort study was conducted at a single post-acute hospital between April 2017 and October 2021 on patients who were admitted for rehabilitation following THA and TKA. The inclusion criteria were patients aged ≥ 60 years with hip and knee osteoarthritis who underwent primary unilateral total hip and knee arthroplasty at a single acute care hospital. Regarding the severity of osteoarthritis based on the Kellgren–Lawrence scale [16], 62 patients were classified as grade 3 and 189 patients as grade 4 in the THA group, whereas 36 patients were grade 3 and 148 patients were grade 4 in the TKA group. In the acute care hospital, THA and TKA were performed using the same surgical procedures and clinical paths for perioperative management and standardized rehabilitation until discharge to a post-acute hospital. THA was performed using the posterolateral approach, preserving the piriformis, and using non-cemented implants. TKA involved a tri-compartmental, cemented prosthesis, with a medial parapatellar surgical approach. Full weight bearing was allowed during the early postoperative period. The exclusion criteria were as follows: (1) patients who were forbidden to undergo rehabilitation because of severe postoperative complications, including infection, periprosthetic fractures, and hip joint dislocation; (2) those who were transferred to other hospitals to receive intensive treatment for exacerbated comorbid conditions; and (3) those who missed assessments related to physical function at admission or discharge. Consequently, 12 patients were excluded, and 435 patients (251 with THA and 184 with TKA) were included in the study. The study was approved by the Ethics Review Board of Fukuoka Mirai Hospital (approval number: 202104-2).

### 2.2. Post-Acute Rehabilitation Setting

Rehabilitation protocols were planned for each patient by a multidisciplinary team comprising physicians, nurses, physical and occupational therapists, and social workers. All patients participated in individualized physical and occupational therapy for 2 h per day, 7 days a week, totaling 14 h per week. The treatment included (1) 60 to 80 min of physical therapy (e.g., transferring, walking with or without ambulation aids, climbing stairs, equilibrium, joint range of motion, and balance training), as well as muscle resistance training for major muscle groups, with two sets of 10 repetitions at 10 repetition maximum; and (2) 40 to 60 min of individual occupational therapy, including basic ADL, learning to use assistive devices, and safety education. Before discharge, patients received practical training for daily activities such as walking on uneven surfaces, negotiating curbs, and climbing stairs at various heights. The discharge criterion was the ability to perform a series of daily activities independently at home, such as bathing, climbing stairs, and walking outdoors.

### 2.3. Outcome Measures

We collected clinical data on age, sex, body mass index (BMI), presence of comorbidities, cognitive function, ADL, extent of pain, and physical function. All measures at admission were assessed within 3 days of hospitalization. The Charlson Comorbidity Index (CCI) was used to assess general comorbidity [17] as it is a useful tool for predicting outcomes in older and comorbid patients. Cognitive functions were assessed using the Mini-Mental State Examination (MMSE). Knee isometric extension strength, which is strongly associated with gait ability in both THA and TKA, was measured using a handheld dynamometer (Anima, Tokyo, Japan) for both the operated and non-operated sides at admission, by positioning the patient on a platform in a sitting position with 90° hip and knee flexion. The strength was measured twice, and the maximum value was used for the analysis.

Functional status was assessed at admission and discharge using the motor component of the Functional Independence Measure (M-FIM), which includes 13 parameters of ADL and mobility. The reliability and validity of the M-FIM have been confirmed [18]. In a previous study, satisfactory functional gain in inpatient rehabilitation was defined as 40% for THA and 45% for TKA using the Montebello Rehabilitation Factor Score (MRFS), which is defined as the M-FIM score change (discharge M-FIM score−admission M-FIM score) divided by the maximum M-FIM score (i.e., 91 points) minus the M-FIM admission score [5].

Pain intensity during walking was measured using a Numerical Rating Scale (NRS) at both time points. Previous research has established that pain intensity using NRS has good to high reliability and validity [19]. To achieve a minimally clinically important difference (MCID) in pain, a patient had to achieve either a reduction of two points on the NRS pain scale or report an NRS pain score of 0, which was used in patients undergoing lower-limb total arthroplasty [19,20].

Gait and mobility were evaluated using the 10 Meter Walk Test (10 MWT) and TUG test at both time points. For the 10 MWT, a 20 m path was used, and the initial and final 5 m of the 20 m path were marked for the acceleration and deceleration phases of the test. This test is very reliable in patients undergoing total lower-limb arthroplasty [21]. A previous report suggested a substantial meaningful change of 0.13 m/s in older adults [22]. The TUG test consisted of standing up from an armless chair, walking in a straight line for 3 m, turning around, and returning to the seated position [23]. Some previous studies have reported that the TUG test had excellent validity and reliability among patients with THA and TKA [24]. The MCID on the TUG test in patients with hip osteoarthritis is 1.4 s [25]. In addition, a 12s score on the TUG test has recently been reported as the new cut-off value for increased fall risk among community-dwelling older adults [26].

Balance function was evaluated using the Berg Balance Scale (BBS), a 14-item balance assessment tool, at admission and discharge, whereby each item was scored on a 0–4 scale, with a total maximum score of 56 [27]. The BBS is considered a valid and reliable test of balance function in patients undergoing lower-limbed arthroplasty [28]; the MCID based on an anchor-based study was reported to be 5 points [29].

### 2.4. Statistical Analysis

Descriptive data are presented as the mean ± standard deviations, numbers, or percentages. The normality of the distribution was assessed using the Shapiro–Wilk test. The significance of the differences in demographic and clinical characteristics between the two groups was statistically analyzed using the unpaired *t*-test for numerical data and the chi-squared test for categorical variables. The paired *t*-test was used to examine differences in clinical outcomes during inpatient rehabilitation after both procedures. Two-way multivariate analysis of covariance (MANCOVA) controlled for age, BMI, and MMSE was used to identify possible main effects considering the dependent variables (ADL, pain, mobility, and balance parameters) and factors such as time (admission to discharge), group (THA and TKA), and interaction time × group. When the MANCOVA identified statistical significance, two-way repeated-measures ANCOVA controlled for the above three factors was performed for each dependent variable. The effect sizes (ES) were calculated using Cohen’s *d* to quantify the magnitude of difference from preoperative to postoperative within each group, considering an ES of 0.19 or less as trivial, 0.20 to 0.49 as small, 0.50 to 0.79 as moderate, and 0.80 or more as large [30]. Multiple linear regression analysis was used to determine predictors associated with improvement in the TUG test for each patient following THA and TKA by focusing on the improvement in comprehensive mobility function ability after surgery. Age, sex, BMI, CCI, MMSE value, M-FIM score, NRS score, knee extension strength on both sides, 10 MWT results, TUG test results, and the BBS score were used in the model for each procedure. Statistical analyses were performed using the JMP^®^ version 16.0 (SAS Institute Inc., Cary, NC, USA). A *p* value < 0.05 was considered statistically significant.

## 3. Results

### 3.1. Baseline Characteristics of Patients with THA and TKA

**Figure 1:**
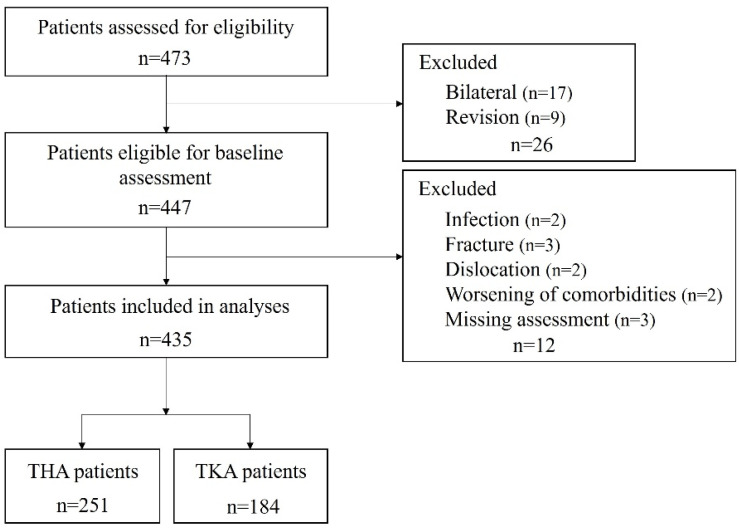
Patient enrollment flow diagram. Abbreviations: THA, total hip arthroplasty; TKA, total knee arthroplasty.

A flow diagram of the study population is presented in Figure 1. The baseline patient characteristics are listed in Table 1. The average ages at admission for patients with THA and TKA were 71.1 and 75.5 years, respectively. Patients who underwent THA were younger (*p* < 0.001), had a lower BMI (*p* < 0.001), and had higher MMSE scores (*p* < 0.001) than those who underwent THA. All patients in this study were discharged directly to home from the post-acute institution.

### 3.2. Clinical Outcomes and Statistical Significance of Dependent Variables

**Figure 2:**
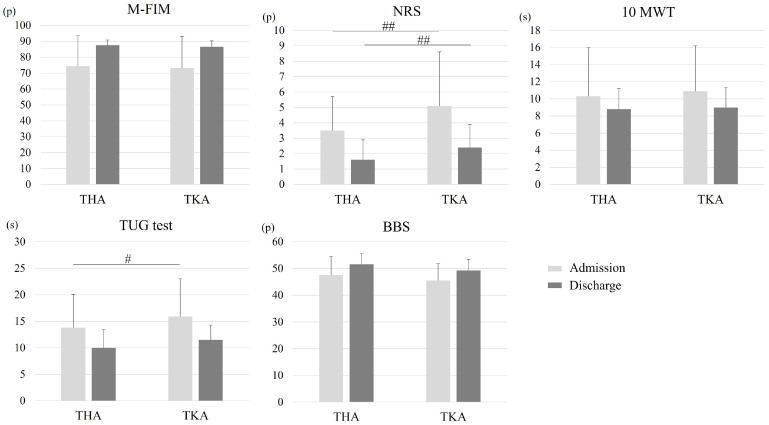
Performance on five outcome measures at admission and discharge for each group. Abbreviations: THA, total hip arthroplasty; TKA, total knee arthroplasty; M-FIM, motor component of the Functional Independence Measure; NRS, Numerical Rating Scale; 10 MWT, 10 M Walk Test; TUG, Timed Up and Go; BBS, Berg Balance Scale; p, points; s, seconds. ^#^ THA vs. TKA at admission (*p* = 0.004). ^##^ THA vs. TKA at admission and discharge (*p* < 0.001).

The mean scores at admission and discharge, mean difference, and associated effect size on the five outcome measures in each group are presented in Table 2. The NRS scores at both admission and discharge were higher in patients with TKA than in those with THA (*p* < 0.001, *p* < 0.001, respectively) (Figure 2). The TUG time was longer in patients with TKA at admission; however, no significant difference was observed at discharge between the two groups (Figure 2). The MANCOVA showed a significant main effect of time, group, and interaction time × group (Table 3). Repeated measures ANCOVA showed a significant main effect of time for all five outcome measures (Table 4). A main effect of the group was obtained with regard to pain, and a main effect of the time x group interaction was also observed for the NRS and TUG test (Table 4).

### 3.3. Clinical Interpretation of Dependent Variables

The mean relative functional gain on the M-FIM score, calculated using the MRFS, was 53.3% in THA and 56.4% in TKA. Satisfactory functional gain, which achieved 40% and 45% of rehabilitation potential, was obtained in 191 patients (76%) with THA and 132 (72%) with TKA during inpatient rehabilitation. Patients who achieved a satisfactory functional gain during post-acute rehabilitation in THA and TKA scored significantly lower on their admission functional ability using M-FIM measurements (*p* < 0.001, *p* < 0.001). For the NRS, 189 patients (75.3%) with THA achieved an MCID (163 with a reduction of two points on the NRS and 26 with an NRS pain score of 0 at discharge). Of the patients with TKA, 136 (73.9%) achieved an MCID (133 with a reduction of two points on the NRS pain scale and three with an NRS pain score of 0 at discharge). In the assessment of gait function at admission, 153 (61%; cane: 138; rolling walker: 15) and 146 (79.3%; cane: 120; rolling walker: 26), respectively. At discharge, 38 patients (15.1%; cane: 43) with THA and 39 (21.2%; cane: 39) with TKA used assistive devices. Regarding the 10 MWT, 158 patients (62.9%) with THA and 104 (56.5%) with TKA showed a substantial meaningful change of 0.13 m/s at discharge. For the TUG test, 197 (78.5%) and 131 (71.2%) patients demonstrated clinically important improvement that exceeded the MCID of 1.4 s after THA and TKA, respectively. At discharge, 188 patients (74.9%) in the THA group and 97 (52.7%) in the TKA group performed the TUG test in less than 12 s, the cut-off score to identify individuals at increased risk of falls. In the assessment of BBS, 191 patients (76.1%) in the THA group and 129 (70.1%) in the TKA group improved by at least 5 points of the MCID.

### 3.4. Multiple Linear Regression Analysis of Clinical Variables and Changes in the TUG Test

In the multiple linear regression analysis, age (β: −0.264, *p* = 0.009) and TUG test (β: −0.884, *p* < 0.001) at admission were independently associated with TUG time improvement in patients who underwent THA. In patients who underwent TKA, non-operated knee extension strength (β: 0.234, *p* = 0.016) and TUG test results (β: −0.783, *p* < 0.001) were significant predictors for the gain of functional mobility (Table 5).

## 4. Discussion

This study highlights the comparison of the recovery process between THA and TKA in a post-acute inpatient rehabilitation setting and identifies determinants influencing mobility function after each procedure. Our results showed that multidisciplinary inpatient rehabilitation provided significant recovery of physical function within the first few months after surgery in both procedures, despite persistent pain at discharge in patients who underwent TKA. The reasons for severe pain following TKA can be explained by the difficulty of cushioning, swelling, and increased internal pressure, as the soft tissue surrounding the knee joint is relatively thin. In addition, considering that postoperative inflammatory responses are higher in TKA than in THA [31], it is possible that surgical trauma is greater in TKA. Furthermore, in this study, early postoperative functional status in both groups and non-operated knee extension strength after TKA were closely associated with gaining mobility function during the post-acute period.

Our results revealed a similar short-term recovery of physical function between THA and TKA in multidisciplinary inpatient rehabilitation, in agreement with previous reports [4,5,6]. However, some studies have shown better improvement in physical function after THA than after TKA in short-term follow-up [1,2,3]. The reasons for this discrepancy may be explained by the differences in the patients’ backgrounds and rehabilitation contents in each study, and the variability of assessment tools used for physical function. Based on data showing that the functional gains during inpatient rehabilitation were similar between the two surgical procedures [5], baseline patient characteristics may closely influence postoperative functional outcomes.

Regarding pain after lower-limb arthroplasty, many authors have suggested that pain reduction was present earlier in patients who underwent THA than in those who underwent TKA [3,32,33,34]. A previous study using patients’ own evaluations showed that pain relief was reported 7 days after THA, but not until 50 days after TKA [32]. Furthermore, some authors demonstrated that patients who had undergone THA showed earlier improvement in pain from 4 to 6 weeks postoperatively than those who had undergone TKA; however, no significant difference was observed 3 months after surgery, indicating that patients who underwent TKA showed a tendency toward slow initial recovery in the early postoperative period [33,34]. In our study, the extent of pain was also higher at both admission and discharge in patients with TKA than in those with THA. The causes of persistent pain in patients with TKA may be associated with the invasiveness of the surgery and the slower recovery of periarticular tissues because of anatomical factors, such as limited soft tissue coverage. Therefore, postoperative pain management in patients undergoing TKA is crucial for improving rehabilitation and overall outcomes. Multimodal local infiltration analgesia and peripheral nerve blocks have been effective in patients with TKA during the acute postoperative phase [35,36]. In the post-acute period, active physiotherapy interventions, including muscle relaxation exercises, joint mobilization, appropriate use of orthoses, pain management with oral analgesics, and lifestyle guidance, should be considered during inpatient rehabilitation. Additionally, a long follow-up period is necessary because of the slower reduction in pain for several months following the procedure.

We used the TUG test as the main outcome, which is commonly used to provide a performance-based outcome measure in patients with osteoarthritis [3,24,25]. In this study, the TUG times were equivalent between patients with TKA and those with THA at discharge, despite a longer time at admission after TKA. An earlier report demonstrated that patients with TKA showed a greater decline in TUG time up to 3 months after surgery than those with THA; however, there was no significant difference at 6 months in both groups [37]. These characteristics should be considered in the rehabilitation strategies adopted following arthroplasty to provide effective rehabilitation for patients undergoing THA or TKA.

Predicting postoperative ambulatory status can help patients understand their individual goals in ADL and help physical therapists determine program goals. It has been demonstrated that preoperative and early postoperative functions in patients undergoing THA and TKA are closely associated with postoperative functional status [38,39]. In a study on the potential of post-acute inpatient rehabilitation for patients undergoing lower-limb arthroplasty, lower functional ability at admission was the most significant predictor of better functional gain, even though final ability was lower [5,40]. This indicates that patients with poorer functional scores at baseline often experience the greatest gain in physical function because they have greater room for functional recovery. Therefore, we should use this information for improving care planning to improve rehabilitation potential and enhance patient motivation for rehabilitation treatment.

Our results revealed that the TUG time at 3 weeks after surgery was a significant indicator of gait function improvement, consistent with previous reports that the pre- or postoperative TUG test was associated with short-term physical function in lower-limb total arthroplasty [34,41]. The TUG test, which includes several movements frequently performed in daily life, appears to reflect the mobility function performance in patients after THA and TKA at discharge to home. This result suggests that rehabilitation interventions to maintain or improve physical function from the preoperative and early postoperative periods should include exercises, including sit-to-stand training and gait with starts, stops, turns, and obstacles. In contrast, the effect size of the 10 MWT test was smaller than that of the TUG test, and it was not extracted as a predictor of gait function at discharge. This suggests that improvement in walking speed is limited to a few months after surgery, based on investigations in which walking ability and performance-based physical function returned to or surpassed preoperative levels at 3 months and beyond [8,42]. Therefore, it is necessary to understand the significance of evaluation tools and to consider more effective rehabilitation assessments. Recently, mechatronic systems for the rehabilitation of patients with gait disturbances have developed rapidly [43,44,45]. These assistive technology devices may allow patients to increase repetitions and training intensity, thereby maximizing effective intervention time. Thus, incorporating adjuvant technology into rehabilitation protocols may enhance gait function and ADL in older patients following THA and TKA in the near future.

Although researchers have investigated the association between knee extension strength of the operated side and gait function measures, some authors have found that the strength of the non-operated side had a strong effect on postoperative gait function [46,47,48]. A previous study showed that the quadriceps strength of the non-operated side after TKA was predictive of short- to middle-term functional ability [46,47]. Our study also showed that non-operated knee extension strength upon admission was a significant predictor of the gain of mobility function. In patients who underwent TKA, the quadriceps and hamstring muscle strengths were lowest after 3 months and recovered to preoperative levels after 6 months [49]. These results may be explained by the fact that non-operated knee extension strength plays a crucial role in improving gait function as a supporting leg in the early postoperative phase, because operated knee extension strength has not sufficiently recovered. Therefore, we believe that strength training for the non-operated side should be more focused on in rehabilitation programs after TKA. Further studies on non-operated knee strength, as a prognostic factor after TKA, are required to obtain a deeper understanding of short- and mid-term outcomes.

This study has some limitations. First, only short-term rehabilitation outcomes were assessed. Our patients were not followed up with after discharge; therefore, information regarding further functional gain is lacking. Second, no data on preoperative information and physical function were obtained from the acute care hospital. These data are important because preoperative physical status and ADL influence postoperative physical function. Third, we did not investigate the impact of patient expectations and motivation, which are closely associated with improvements in functional outcomes and satisfaction. Fourth, this was a single-center study; therefore, a multicenter study is required to generalize the results. Finally, this study is retrospective and thus it has inherent limitations, such as the availability of outcome measures from patient files and missing data. In some cases, these limitations may have biased our results. Therefore, additional multi-institutional prospective research with a longer follow-up period and the assessment of other factors associated with rehabilitation outcomes are needed to confirm our results.

## 5. Conclusions

Our results indicated that patients with THA and TKA showed similar short-term recovery from multidisciplinary inpatient rehabilitation despite persistent pain at discharge after TKA. Furthermore, the gain in physical function in the post-acute period was independently associated with early postoperative functional levels in both groups and non-operated knee extension strength after TKA. Based on these findings, we emphasize the necessity of strict pain management and muscle strength training on the non-operated side in post-acute rehabilitation programs, especially for patients with TKA. These investigations may be useful for detailed planning and optimization of inpatient rehabilitation program management during the post-acute period.

## Figures and Tables

**Table 1:** Baseline characteristics in patients following THA and TKA.

	THA (n = 251)	TKA (n = 184)	
Characteristic	Mean ± SD	Mean ± SD	*p* Value
Age (years)	71.1 ± 6.9	75.5 ± 6.7	<0.001 ^#^
Female sex *	224 (89.2)	156 (84.7)	0.166
Body mass index (kg/m^2^)	23.3 ± 3.3	25.4 ± 3.6	<0.001 ^#^
Charlson Comorbidity Index (points)	0.4 ± 0.8	0.7 ± 1.1	0.062
Mini-Mental State Examination (points)	27.7 ± 2.6	26.3 ± 4.0	<0.001 ^#^
POD in acute care hospital (days)	17.9 ± 6.7	18.4 ± 4.6	0.385
LOS in post-acute hospital (days)	36.0 ± 22.1	36.7 ± 21.6	0.738
Knee extension strength (kgf/kg) operated	141.1 ± 68.1	93.8 ± 50.9	<0.001 ^#^
non-operated	149.0 ± 64.8	137.1 ± 72.8	0.304

Abbreviations: THA, total hip arthroplasty; TKA, total knee arthroplasty; POD, postoperative day; SD, standard deviation. * Number (%). ^#^ Statistically significant.

**Table 2:** Mean, mean difference, Confidence Interval, and effect size for dependent variables.

	Admission	Discharge	Mean Difference	95% CI for Mean		Effect Size
Outcome Measures	Mean ± SD	Mean ± SD	Mean ± SD	Difference	*p* Value *	Cohen’s d
M-FIM score (points)						
THA	74.3 ± 19.2	87.5 ± 3.3	13.2 ± 6.7	11.9–15.6	<0.001 ^#^	0.96
TKA	73.2 ± 19.8	86.6 ± 3.7	13.4 ± 6.5	11.1–15.2	<0.001 ^#^	0.94
NRS (points)						
THA	3.5 ± 2.2	1.6 ± 1.3	1.9 ± 1.7	1.7–2.2	<0.001 ^#^	0.83
TKA	5.1 ± 3.7	2.4 ± 1.5	2.7 ± 1.6	2.5–2.9	<0.001 ^#^	0.96
10 MWT (seconds)						
THA	10.3 ± 5.7	8.8 ± 2.4	1.5 ± 2.5	1.2–1.8	<0.001 ^#^	0.34
TKA	10.9 ± 5.3	9.0 ± 2.3	1.9 ± 2.0	1.5–2.2	<0.001 ^#^	0.47
TUG test (seconds)						
THA	13.8 ± 6.3	10.0 ± 3.5	3.8 ± 3.6	3.3–4.2	<0.001 ^#^	0.75
TKA	15.9 ± 7.1	11.5 ± 2.8	4.4 ± 3.8	3.9–4.8	<0.001 ^#^	0.82
BBS (points)						
THA	47.5 ± 7.1	51.6 ± 4.1	4.1 ± 3.6	3.7–4.6	<0.001 ^#^	0.71
TKA	45.5 ± 6.4	49.2 ± 4.2	3.7 ± 3.4	3.2–4.2	<0.001 ^#^	0.68

Abbreviations: THA, total hip arthroplasty; TKA, total knee arthroplasty; M-FIM, motor component of the Functional Independence Measure; NRS, Numerical Rating Scale; 10 MWT, 10 Meter Walk Test; TUG, Timed Up and Go; BBS, Berg Balance Scale, SD, standard deviation; CI, Confidence Interval. * Admission to discharge. ^#^ Statistically significant.

**Table 3:** The two-way multivariate ANCOVA for repeated measures.

Effect		Wilks’ λ	F	*p*	η^2^
Time	admission to discharge	0.42	6.47	<0.001 ^#^	0.72
Group	THA versus TKA	0.63	4.04	0.016 ^#^	0.51
time × group		0.54	4.35	<0.001 ^#^	0.54

Abbreviations: ANCOVA, analysis of covariance; THA, total hip arthroplasty; TKA, total knee arthroplasty. ^#^ Statistically significant.

**Table 4:** Main and interaction effects of time and group on 5 outcome measures.

	Time Main Effects	Group Main Effects	
Outcome Measure	(Admission to Discharge)	(THA vs. TKA)	Time × Group Interraction Effects
M-FIM score	*F* = 7.04, *p* < 0.001 ^#^	*F* = 2.85, *p* = 0.051	*F* = 0.89, *p* = 0.345
NRS	*F* = 105.95, *p* < 0.001 ^#^	*F* = 12.28, *p* < 0.001 ^#^	*F* = 8.12, *p* < 0.001 ^#^
10 MWT	*F* = 4.41, *p* = 0.038 *^#^*	*F* = 2.24, *p* = 0.224	*F* = 1.68, *p* = 0.325
TUG test	*F* = 59.78, *p* < 0.001 ^#^	*F* = 2.78, *p* = 0.096	*F* = 6.03, *p* = 0.008 ^#^
BBS	*F* = 5.31, *p* < 0.001 ^#^	*F* = 3.79, *p* = 0.115	*F* = 2.55, *p* = 0.111

Abbreviations: THA, total hip arthroplasty; TKA, total knee arthroplasty; M-FIM, motor component of the Functional Independence Measure; NRS, Numerical Rating Scale; 10 MWT, 10 Meter Walk Test; TUG, Timed Up and Go; BBS, Berg Balance Scale. ^#^ Statistically significant.

**Table 5:** Multiple linear regression analysis between variables at admission and the change of TUG test in patients with THA and TKA.

	Beta	SE	95% CI (Lower–Upper)	*p* Value
THA				
Age	−0.264	0.061	0.045–0.296	0.009 ^#^
TUG test	−0.884	0.104	0.451–0.877	<0.001 ^#^
TKA				
Knee extension strength (non-operated)	0.234	0.005	0.027–0.004	0.016 ^#^
TUG test	−0.783	0.108	0.362–0.808	<0.001 ^#^

Abbreviations: THA, total hip arthroplasty; TKA, total knee arthroplasty; TUG, Timed Up and Go; SE, standard error; CI, Confidence Interval. ^#^ Statistically significant.

## Data Availability

The data that support the findings of this study are available from the corresponding author upon reasonable request. Neither the patients nor the public were involved in the design, conduct, reporting, or dissemination plans of our research.
